# Temperature and Dissolved Oxygen Drive Arsenic Mobility at the Sediment—Water Interface in the Lake Taihu

**DOI:** 10.3390/toxics12070471

**Published:** 2024-06-29

**Authors:** Liqing Zeng, Fan Yang, Yuyan Chen, Songmei Chen, Mei Xu, Chongyu Gu

**Affiliations:** 1 Department of Public Health and Medical Technology, Xiamen Medical College, Xiamen 361021, China; 202206520063@xmmc.edu.cn (Y.C.); 202206520033@xmmc.edu.cn (S.C.); 202206520047@xmmc.edu.cn (M.X.); 202206520006@xmmc.edu.cn (C.G.); 2 Key Laboratory of Urban Environment and Health, Institute of Urban Environment, Chinese Academy of Sciences, Xiamen 361021, China; fyang@iue.ac.cn

**Keywords:** arsenic, temperature, dissolved oxygen, sediment—water interface, redox transformation

## Abstract

In this study examining the effects of temperature and dissolved oxygen (DO) on arsenic (As) release at the sediment–water interface (SWI), it was found that an increase in temperature promoted the formation of an anaerobic environment and the reduction and desorption of As fractions within the sediments. A temperature of 32 °C was the most favorable condition for As release at the SWI, and low DO conditions aggravated this process. Even under high DO conditions, the release of sediment As was significantly accelerated under high-temperature conditions, allowing dissolved As to rapidly migrate to the overlying water. In this process, the release of As from sediments was a consequence of the transformation of As fractions in the sediments.

## 1. Introduction

Arsenic (As) is a common element found in the Earth’s crust, albeit in relatively low amounts. However, industrial activities and population growth have resulted in substantial contamination of the terrestrial environment [1]. Exposure to As can cause symptoms like nausea and vomiting, and in severe cases, it can be fatal. Chronic As exposure is associated with liver and kidney damage, as well as an increased risk of cancer [2]. The World Health Organization emphasizes that As contamination, often due to watershed development and industrial pollution, is a significant threat to freshwater ecosystems and a major challenge to global drinking water safety [3]. 

Shallow lakes are unique aquatic systems where water and sediments interact frequently. In these sediments, As predominantly occurs as As(V) and As(III), and their transformation and mobility are highly sensitive to environmental conditions at the SWI. This has been a focal point of scientific research for the past few decades [4,5]. Fabian et al. (2003) studied the cycling of As in the sediments of Lake Baldegg, a eutrophic lake in Switzerland, and explained how different environmental conditions, including redox status, microbial activity, and organic matter, affect the transformation of As species [6]. Schuh et al. (2018) analyzed how historical gold smelting activities in the Yellowknife region of Canada have influenced the speciation and mobility of As in lake sediments, emphasizing the effects of human activities on As stability and movement [7]. Couture et al. (2010) created and used a non-steady-state model to simulate the diagenesis of As in lake sediments, with a focus on predicting how As transformations occur under changing environmental conditions [8]. Despite the valuable insights provided by these studies, a systematic controlled investigation of the influence of temperature and dissolved oxygen (DO) on As mobility and transformation in sediments of shallow lakes remains underexplored.

Temperature fluctuations in aquatic environments can affect sediment properties, microbial activity, and the speciation of As, thereby influencing the migration behavior of sediment-bound As [9]. Temperature changes can also impact non-biological processes like adsorption and precipitation of As in sediments [10,11,12]. Lower temperatures reduce the activity space for molecules at the adsorption sites, which makes it more favorable for adsorbing additional molecules onto the solid surface. Higher temperatures can facilitate the physical desorption of As, causing it to migrate into the aqueous phase. Adsorption processes encompass various types, including physical, chemical, and biological adsorption, as well as precipitation and chelation reactions, all of which are associated with activation energy. Several factors influence the adsorption process, and they interact with one another [13]. Sediment properties may exert a greater influence on the adsorption process than temperature alone [14]. For instance, some studies have demonstrated that increasing temperature reduces arsenate adsorption, while others have found that solid-phase adsorption of As increases with rising temperature [15,16]. Therefore, temperature variations not only impact the associated biogeochemical processes of sediment-bound As but also significantly affect the physical adsorption–desorption processes that govern its release. Therefore, it is crucial to investigate the impact of temperature on the migration of As associated with sediments in shallow lakes, where there are significant temperature variations and pollution.

Under both low and high DO conditions, various fractions of sediment-bound As can undergo changes that lead to its release [17]. In shallow lakes, wind and waves can maintain the sediment–water interface (SWI) at aerobic levels, reducing the rate of As release. However, during the summer season, when winds are minimal and oxygen demand is high, the surface sediments may become anaerobic. Alternating aerobic and anaerobic conditions cause variations in redox potential. Currently, studies tend to focus on examining the influence of DO or temperature on sediment As release individually, with fewer exploring the combined effects of both factors. Therefore, this study aims to investigate the influence of temperature and DO on As release at the SWI and to understand the mechanisms underlying As release from sediments under different temperature and DO conditions.

Factors such as geographical location, water characteristics, and sediment properties can contribute to variations in the mechanism of temperature and DO in different environments. Nonetheless, field investigations and experimental studies that primarily focus on the isolated effects of temperature or redox potential fluctuations [18,19] offer relatively limited insight into how variations in temperature and DO impact As release in shallow lake sediments during the cultivation process, especially in relation to the transformation of different As species. Therefore, further exploration of specific mechanisms is needed to enhance our understanding of the precise role they play in regulating As mobilization between surface sediments and water in natural shallow water or aquatic systems under varying temperatures and DO environmental conditions.

Taihu Lake, the second-largest freshwater lake in China, plays a crucial role in the Yangtze River Delta region by providing fresh water, supporting agriculture, facilitating water resource development, and enabling navigation [20]. The rapid industrial and agricultural development over the past few decades has resulted in severe metal pollution in the waters of Lake Taihu [21]. The total As concentration in the sediments exceeds the environmental background value and is higher than that in many regions worldwide [22]. The total soluble As concentration in drinking water once surpassed the guideline standard of 10 μg/L set by the World Health Organization (WHO) [23]. This poses a serious threat to environmental safety and human health. The water and sediment environment of the lake exhibits significant fluctuations across different seasons [24]. Seasonal changes in the lake’s ecological environment may alter the bioavailability and the release process of As in the sediments, thereby leading to As pollution in the water [25]. Therefore, this study aims to explore the differences in As release behavior at the SWI of Lake Taihu under varying temperature and DO levels, thereby clarifying the relationship between the migration of different forms of As in the sediments and environmental changes, and offering both technical and theoretical reference for the surveillance and remediation of As contamination in shallow lake ecosystems.

## 2. Materials and Methods

### 2.1. Sample Collection and Preparation

In January 2016, field samples were obtained from Zhushan Bay in Lake Taihu (located at coordinates N 31°22′53″, E 120°03′11.5″) for incubation. Water samples and surface sediment samples were drawn from depths less than 0.50 m and from about 10 cm depth with a grab sampler. To ensure the homogeneity of the sediment samples, they were extensively blended and then enclosed in airtight, clean polyethylene bags, preventing any air from being enclosed. These samples were then kept in a cold and dark environment until they were sent to the laboratory. Upon arrival at the laboratory, the sediments samples were subjected to freeze-drying and grinding to sieve by a 1.0-mm mesh nylon sieve for the subsequent indoor incubation and by a 100-mm mesh nylon sieve for the characterizations and As chemical fractions of the sediment, respectively. These processes were used in our previous study [26].

### 2.2. Experimental Mesocosms

At room temperature, 12 cm of fresh sediment was evenly added to a transparent Plexiglass tube (with a diameter of 7 cm and a length of 50 cm), was sealed at the bottom with rubber plugs, and was wrapped in aluminum foil to avoid light. Subsequently, water samples were gradually added to the tube until the height of the water column reached a height of 30 cm. Each treatment group was set up with two replicates. These tubes were incubated in a plastic box containing 42 cm of lake water. After reaching equilibrium for two weeks, a new SWI was formed [27]. According to the seasonal temperature change of Taihu Lake, incubation temperatures of 5 °C, 20 °C, and 32 °C were set and incubated. For high DO groups, an air pump was employed to aerate the overlying water continuously for 8 h daily, ensuring a range of 5 mg/L < DO < 7 mg/L. Low DO groups were filled with nitrogen with a purity of 99% in the overlying water. The nitrogen was filled at a rate of 2 L/min in the morning and evening every day, each time for 2 h. Following nitrogen filling, vaseline was used for sealing treatments to maintain DO < 1 mg/L.

The experiment spanned a period of 30 days, during which samples were collected on the 1st, 2nd, 6th, 17th, and 30th days. Water samples were passed through a 0.45 μm cellulose acetate membrane for filtration. Following this, the sediment samples were sectioned into 2 cm segments, centrifuged at 5000 rotations per minute for 15 min, and then subjected to filtration through a 0.45 μm cellulose acetate membrane to isolate the pore water. These samples were then preserved at −20 °C for elemental analysis [27]. Additionally, Eh variations in sediments were continuously observed. During the incubation, the level of surface water was kept constant, with any decreases due to evaporation or other factors being promptly replenished with lake water.

### 2.3. Sampling and Analysis

The physicochemical property of water was monitored with a water quality analyzer Multi 3420 (WTW, Xylem Inc, Weilheim, Germany). The CO_2_-free deionized water and the sediment were homogeneously mixed in a ratio of 1:2.5, agitated for 30 min at 250 rpm, and then centrifuged at 4000 rpm for 10 min to measure the pH value of the sediment [28]. The moisture content within the sediment was measured according to the method described by Lu et al. [29]. The sediment profiles’ pH and Eh values and total organic carbon (TOC) were quantified with the aid of microelectrodes (Unisense, Denmark) and a TOC analyzer (Elementar, German), respectively. 

As, Mn, and Fe concentrations in the superjacent and pore waters were quantified via an inductively coupled plasma mass spectrometer (ICP-MS, Agilent7500cx, Agilent Technologies, Santa Clara, CA, USA). The helium mode was employed during the measurements to mitigate potential interferences from ^40^Ar and ^35^Cl. The reliability of the ICP-MS apparatus was ensured by monitoring with internal standards containing ^115^In and ^103^Rh. The concentrations of ferrous and sulfide ions in the water were quantified by the ortho-phenanthroline colorimetric method [30] and the methylene blue method [31], respectively.

According to the method used by Zhu et al. [32], the As (V) and As (III) species were quantified via a high-performance liquid chromatography (HPLC, Agilent1200, Agilent Technologies, Santa Clara, CA, USA) jointed with an ICP-MS (Agilent LC1100, Agilent Technologies, Santa Clara, CA, USA). An anion exchange column was used for separation, employing a mobile phase composed of 10 mmol/L NH_4_H_2_PO_4_ and 10 mmol/L NH_4_NO_3_, which was adjusted to pH 6.2 with HNO_3_ or ammonia solution. An X-ray fluorescence spectrometer (XRF, model AxiosmAx; PANalytical, Almelo, The Netherlands) was used to measure As, Mn, Fe, and S in the sediment. A sequential extraction protocol, as described by Wang et al. [33]. was conducted to discern the various As forms in the sediment, including water-soluble As, exchangeable As, amorphous iron-bound As, crystalline iron-bound As, organic-bound As, and residual As. The fractions F3 and F4 representing FeOOH and MnOOH, respectively, and were extracted simultaneously.

### 2.4. Quality Assurance and Data Analyses

Standard substances were used to maintain the accuracy and reliability of sample analysis. A reference substance, GSD-9, served as a baseline for quality control during the determination of total elements and As fraction in sediments. Standards like As (GSB 07-1714-2004) and Mn (GSB 04-1736-2004) were utilized to calibrate the levels of As and Mn measured in water. FeSO_4_·(NH_4_)_2_SO_4_·6H_2_O and Na_2_S were used as standard regents of ferrous and sulfide solution, respectively. The standard substances of As (V) and As (III) were prepared from Na_3_AsO_4_·12H_2_O (Fluka) and NaAsO_2_ (Alfa Aesar), respectively. LODs of the measurement methods, the linear equation of the standard regression curve of each element, and the correlation coefficient are presented in Table S1.

Regents with analytical grade used in the experiments (National Pharmaceutical Group Chemical Reagent Co., Ltd. Shanghai, China) were kept at −20 °C, thawed, and diluted as necessary for constructing standard curves. The analysis accuracy was evaluated with a recovery rate of 95–106% (*n* = 4) for the standard, and the relative standard deviation was kept within ±5%. Each sample was measured thrice to maintain an analysis error below 10%.

Prior to use, the glassware and centrifuge tubes underwent a 48-h soak in a 10% solution of high-purity HNO_3_, followed by a thorough rinse with ultrapure water.

The cumulative release rate of each element was calculated by the formula as follows [34]:

(1) $$r=\frac{{V(C}_{n}-C_{0})}{{(t}_{n}-t_{0})A}$$

The accumulative release rate of an element, denoted as *r* (μmol/(m^2^·d)), is calculated using the concentration of the element on the nth day (*C_n_*, μmol/L) in the overlying water, the initial concentration of the element (*C*_0_, μmol/L), the surface area of the SWI (*A*, m^2^), and the volume of the overlying water (*V*, L). *t_n_* and *t*_0_ express as the nth day of the experimental time and the initial time, respectively.

The temperature coefficient (*Q*_10_), quantifying the impact of temperature on metabolic processes [35], was employed to assess the influence of temperature on the reduction rates of Mn, Fe, and sulfide, as well as the release rate of As in the sediments. The formula is shown in the following formula:

(2) $$Q_{10}={\left(\frac{r_{2}}{r_{1}}\right)}^{\left(\frac{10}{T_{2}-T_{1}}\right)}$$

where *r*_2_ and *r*_1_ represent the release rates of elements at *T*_2_ and *T*_1_, respectively, *T*_1_ < *T*_2_ (°C).

All experimental results were analyzed using Microsoft Excel 2013 and SPSS 20.0. Duncan’s multiple-range test was utilized in one-way ANOVA to identify significant differences among the groups. Pearson correlation analysis was conducted to examine the relationships between variables, with a *p*-value less than 0.05 indicating statistical significance. The quantification of As species content was achieved by integrating the peak areas of each species using Origin 8.5 software.

## 3. Results and Discussion

### 3.1. The Influence of Temperature Variations on the Redox Environment of Sediments under Different DO Levels

**Figure 1:**
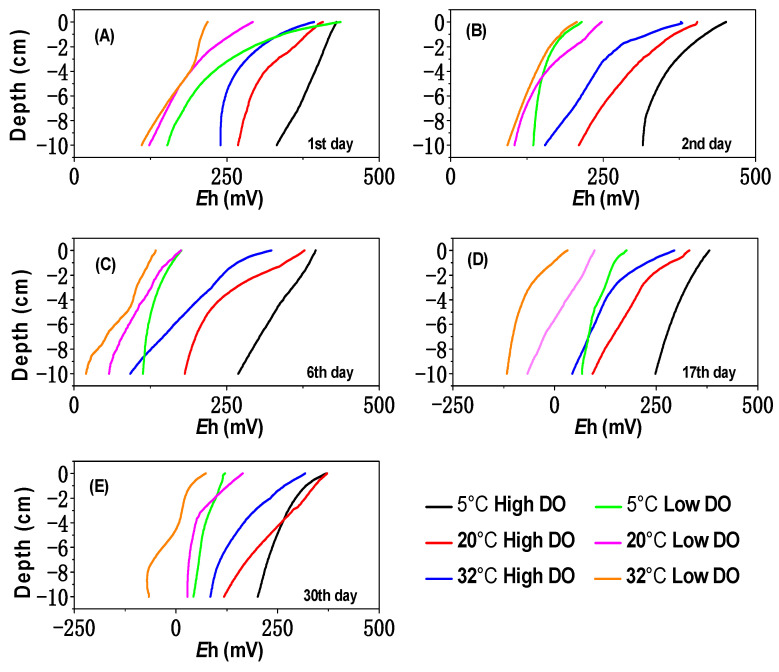
The effect of temperature on Eh of sediments under differing DO conditions on (**A**) 1st day, (**B**) 2nd day, (**C**) 6th day, (**D**) 17th day, and (**E**) 30th day.

The variations of Eh values over time in different treatment groups are depicted in Figure 1. On account of the high organic matter content in Zhushan Bay of Lake Taihu [26], microbial oxygen demand during organic matter degradation was substantial, resulting in rapid consumption of DO in the pore water. The influence of temperature on this process was significant. Under high DO and 5 °C conditions, Eh slowly decreased, with an average value of +258 mV at the end of the incubation period. This can be attributed to lower microbial activity and lower DO demand under low-temperature conditions. At 20 °C, Eh decreased more noticeably and reached its lowest value on the 17th day, with an average of +187 mV. Under 32 °C, Eh significantly dropped to +126 mV on the 17th day, which might be due to the optimal temperature for microbial growth and maximum microbial activity, leading to higher oxygen demand and faster depletion of DO.

During the low DO incubation period, sediment Eh in all temperature conditions showed varying degrees of decrease. As shown in Figure 1, compared to low-temperature conditions, sediment Eh decreased more significantly under high-temperature conditions, reaching a strongly reducing condition more quickly. On the 17th day, the average Eh values for the 5 °C, 20 °C, and 32 °C treatment groups under low DO conditions were +105 mV, +33 mV, and −73 mV, respectively. Subsequently, the 5 °C treatment group continued to decline to +73 mV, while the 20 °C and 32 °C treatment groups slowly rose to +60 mV and −16 mV, respectively. Therefore, temperature variations had a significant impact on the changes in Eh under different DO conditions.

### 3.2. Effect of Temperature on Dissolved Fe, Mn, and S under Different DO Conditions

Figures S1–S3 illustrate the fluctuations of dissolved Fe, Mn, and S concentrations in sediment pore water under various cultivation conditions. Specifically, Figure S1 depicts the impact of temperature on the content of dissolved Fe in pore water across different DO levels; Figure S2 highlights the effect of temperature on the content of dissolved Mn in pore water under a range of DO concentrations; and Figure S3 demonstrates the influence of temperature on the dissolved S content in pore water under diverse DO conditions. Notably, the release of dissolved Fe and Mn into the pore water exhibited a pronounced temperature dependence.

Under 5 °C conditions, both high and low DO cultivations showed a slow decrease in sediment Eh (Figure 1), resulting in a slow release of dissolved Fe and Mn in the pore water. After 30 days, the average dissolved Fe concentrations were 0.96 mg/L and 1.87 mg/L, while those of dissolved Mn concentrations were 3.87 mg/L and 6.13 mg/L in the high and low DO cultivations, respectively. As the temperature increased, the DO concentration in the water decreased, leading to a decrease in Eh (Figure 1). This intensified the reduction reaction of Fe/Mn-OOH in the sediments, resulting in a significant increase in dissolved Fe and Mn in the pore water. Under 32 °C conditions, there was a noticeable enhancement in the concentrations of dissolved Fe and Mn in the pore water. The maximum average values of dissolved Fe were 5.90 mg/L and 6.34 mg/L in the high and low DO environments, respectively. The maximum average values of dissolved Mn were 9.53 mg/L and 12.10 mg/L in the high and low DO environments, respectively. These values were significantly higher than the maximum average values of dissolved Fe and Mn at 5 °C under the conditions of high and low DO (*p* < 0.05) (Figures S1 and S2). Additionally, the peak positions of dissolved Fe and Mn in the profile of sediment became shallower (Figures S1 and S2), shifting from −6 to −10 cm at 5 °C to −4 to −6 cm at 32 °C. The significant release of sediment Fe and Mn under high-temperature conditions might be attributed, on the one hand, to the greater favorability for desorption of Fe and Mn at high temperatures [16,36], and on the other hand, to the enhanced activity of sediment Fe and Mn reducing bacteria under low DO conditions at 32 °C. This further enhanced the process of reducing and dissolving Fe/Mn-OOH. After 30 days of cultivation, there was a varying degree of decline in the amounts of dissolved Fe and Mn in the pore water at 20 °C and 32 °C. Under high DO environments, dissolved Fe (Mn) dropped to 1.82 mg/L (6.23 mg/L) and 2.12 mg/L (5.04 mg/L) at 20 °C and 32 °C, respectively. In sediments under low DO environments, the soluble Fe (Mn) at the corresponding temperature conditions also decreased to 4.05 mg/L (8.65 mg/L) and 2.23 mg/L (8.07 mg/L), respectively. This might be due to the complexation, adsorption, coagulation, and co-precipitation of released Fe and Mn ions with other leachates, leading to their decline [37]. It could be observed that there was a significant decrease in the amounts of soluble Fe and Mn under high-temperature cultivation at 32 °C (Figures S1 and S2). This could potentially be associated with the facile generation of secondary minerals or colloids of dissolved Fe and Mn under high-temperature conditions [38,39].

**Figure 2:**
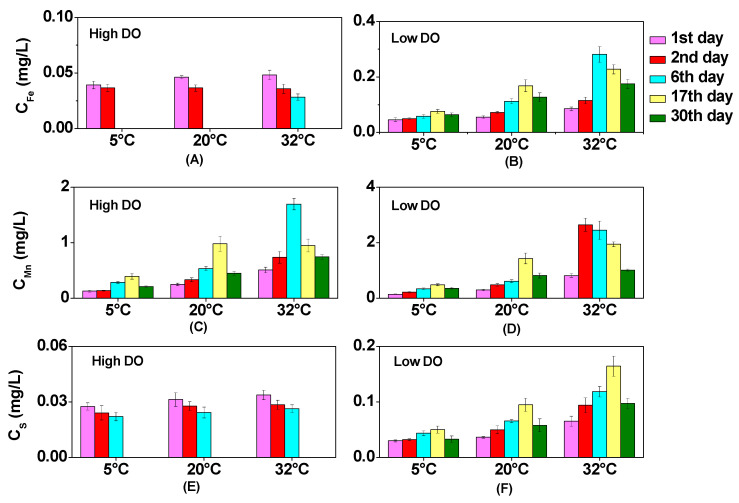
The impact of temperature on dissolved Fe, Mn, and S in overlying water under varying DO conditions: high DO: (**A**) Fe, (**C**) Mn, and (**E**) S; low DO: (**B**) Fe, (**D**) Mn, and (**F**) S.

The overlying water exhibited fluctuations in its dissolved Fe and Mn content (Figure 2). Under high DO cultivation, the inhibitory effect of DO on the dissolved Fe became apparent in the overlying water, with dissolved Fe being oxidized to levels below the detection limit on the 2nd or 6th day. Although oxygen input could lead to the gradual oxidation of Mn(II) to less soluble forms such as Mn(III) [40], the oxidation potential of Mn remained high. Therefore, a relatively high concentration of Mn (II) still persisted in the overlying water throughout aerobic cultivation (Figure 2). After high DO cultivation, the contents of dissolved Fe (Mn) at 5 °C, 20 °C, and 32 °C decrease to 0.11 mg/L (0.84 mg/L), 0.06 mg/L (1.09 mg/L), and 0.07 mg/L (1.53 mg/L), respectively, in the overlying water. These results may be related to the complex biogeochemical processes occurring within the sediment and its interaction with the overlying water.

Low DO conditions were favorable for the reduction reaction of Fe/Mn-OOH [41], leading to an evident enhancement in the levels of dissolved Fe and Mn in the overlying water (Figure 2). After cultivation, the contents of dissolved Fe in the overlying water at 5 °C, 20 °C, and 32 °C were 0.064 mg/L, 0.13 mg/L, and 0.18 mg/L, respectively, whereas the concentrations of dissolved Mn were 0.36 mg/L, 0.82 mg/L, and 1.01 mg/L, respectively. As the temperature increased, there was a corresponding increase in the migration rate of dissolved Fe and Mn from the pore water to the overlying water. Therefore, the maximum concentrations of dissolved Fe and Mn in the overlying water were observed earliest at 32 °C (Figure 2). The peak value of dissolved Fe reached 0.28 mg/L on the 6th day under the condition of low DO, while the peak values of dissolved Mn were 1.70 mg/L on the 6th day under high DO conditions and 2.65 mg/L on the 2nd day under low DO conditions.

Under aerobic conditions, sulfides were easily oxidized by DO and Fe/Mn-OOH to form sulfates, which inhibited the diffusion of soluble sulfides to the upper layers of the sediment [42,43]. Therefore, the content of soluble sulfides in the upper pore water (0–2 cm) was low during high DO cultivation (Figure S3), and the content of soluble sulfides in the overlying water also decreased (Figure 2). Under high DO conditions, the increase in temperature facilitated the escape of DO, coupled with the continuous consumption of DO by organic matter, leading to a gradual decrease in Eh (Figure 1). There was a slight boost in dissolved S content of the pore water at 20 °C and 32 °C, reaching maximum values of 0.11 mg/L and 0.18 mg/L on the 17th day, respectively. Previous studies have shown that sulfate-reducing bacteria have the ability to reduce sulfates when Eh was less than +100 mV, and under conditions of 25–35 °C, these bacteria were more active, resulting in more soluble sulfides during the reduction of sulfates [44]. This could result in an increase in the dissolved S in sediments under high DO and high-temperature conditions.

In anaerobic environments, the activity of sulfate-reducing bacteria had a more significant impact on the reduction of sulfates in sediments [45], and the maximum content of soluble S in the pore water under low DO conditions in the 5 °C, 20 °C, and 32 °C treatment groups were 3.1 times, 2.5 times, and 2.3 times higher than that under high DO conditions at the corresponding temperature conditions (Figure S3). Under low DO cultivation, the Eh of the sediment continued to decrease, but the content of soluble sulfides in the pore water decreased from the 17th day at 20 °C and 32 °C, which was inconsistent with the continuous increase in the concentration of soluble sulfide at 5 °C. This was likely because sulfide precipitation occurs under stronger anaerobic conditions at 20 °C and 32 °C, resulting in a decline in the content of soluble sulfide [46]. Under low DO cultivation, the content of dissolved sulfide at 5 °C showed little change (0.030–0.050 mg/L) in the overlying water. The higher temperature promoted sulfate reduction and the production of soluble sulfide, leading to an increase in the content of dissolved sulfide. On the 17th day, at 20 °C and 32 °C, the content of dissolved sulfide in the overlying water increased from 0.036 mg/L and 0.065 mg/L, respectively, to 0.095 mg/L and 0.16 mg/L.

### 3.3. The Change of Dissolved As in Sediments

**Figure 3:**
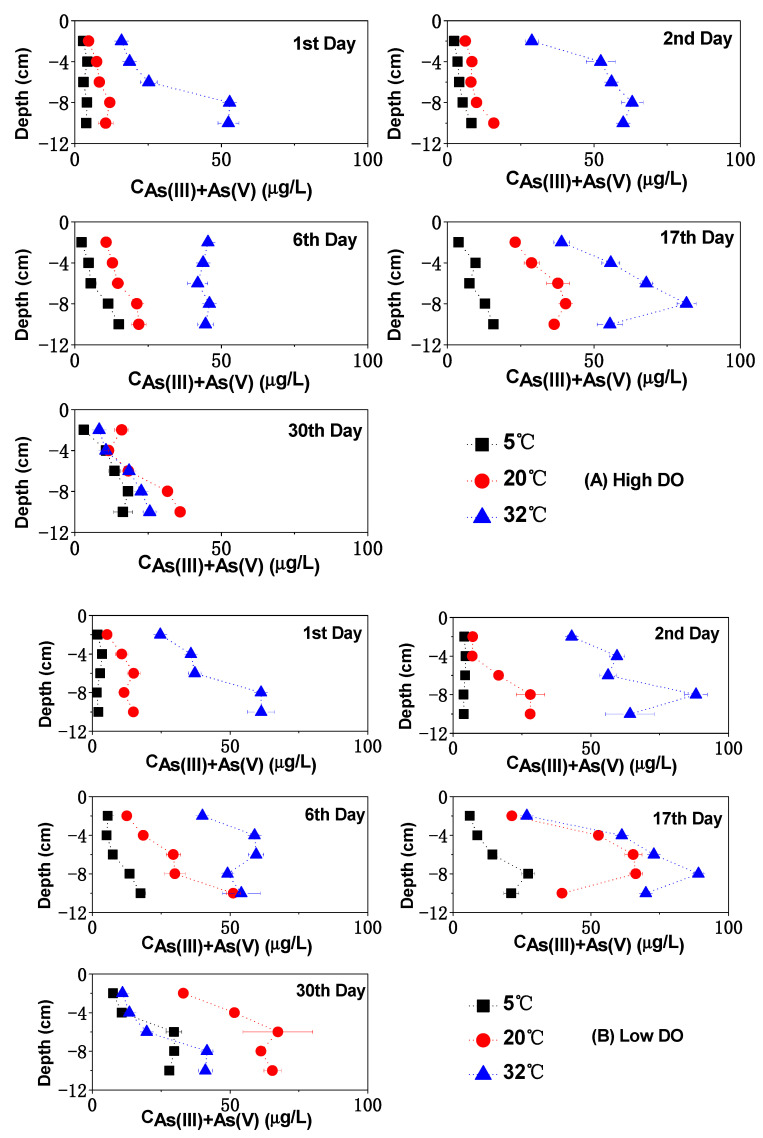
The impact of temperature on As (III) + As (V) concentration in the pore water under different DO conditions: (**A**) high DO, (**B**) low DO.

**Figure 4:**
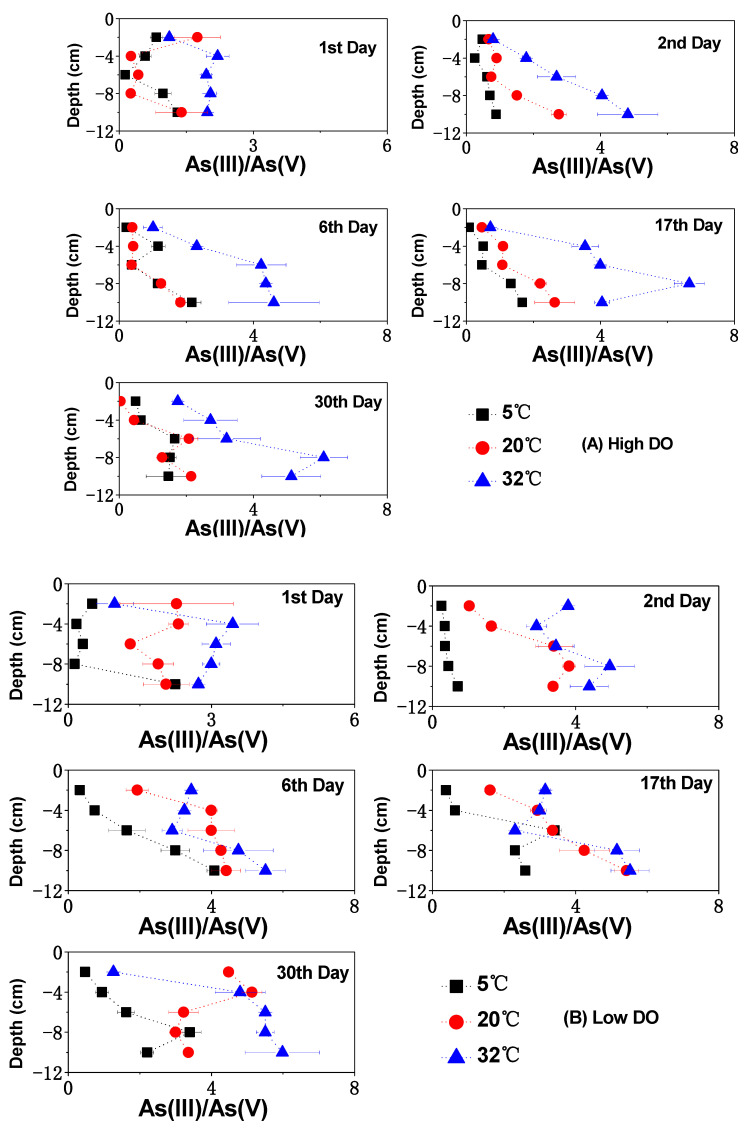
The impact of temperature on As (III)/As (V) ratio of pore water under different DO conditions: (**A**) high DO, (**B**) low DO.

**Figure 5:**
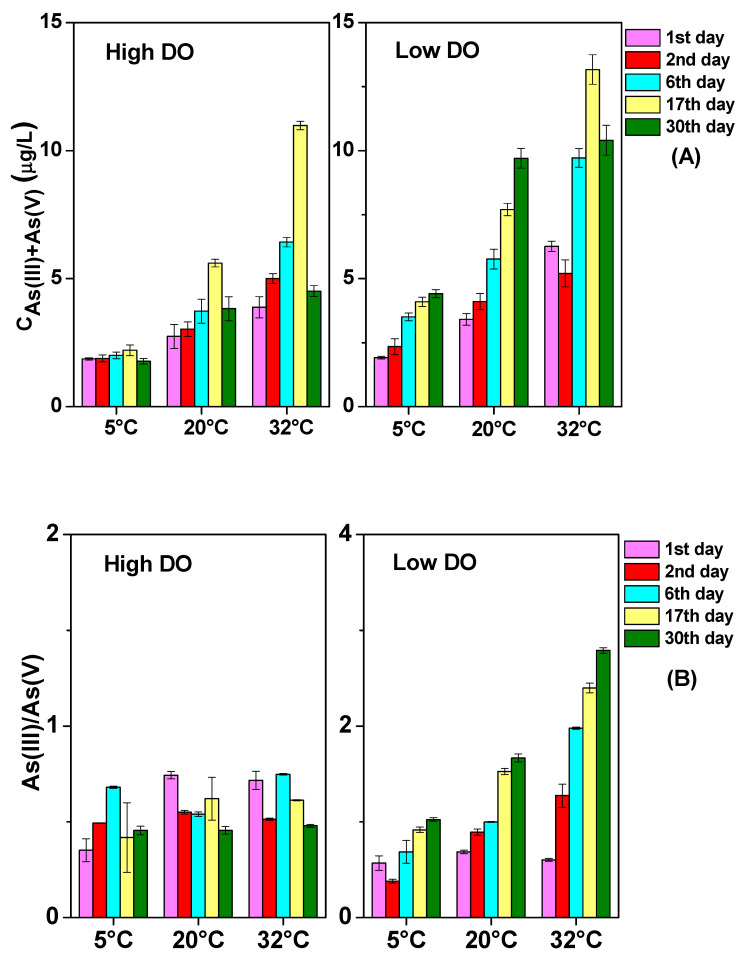
The impact of temperature on As (III) + As (V) content (**A**) and As (III)/As (V) (**B**) of superjacent water under different DO conditions.

The changes in soluble As concentrations in the pore water and overlying water under different DO and temperature conditions are shown in Figure 3, Figure 4 and Figure 5. Under the 5 °C condition, after high and low DO incubation, the average contents of soluble As (III) + As (V) in the pore water were 12.40 μg/L and 21.14 μg/L, with corresponding average ratios of As (III)/As (V) of 1.16 and 1.73. The contents of soluble As (III) + As (V) in the overlying water were 1.77 μg/L and 4.41 μg/L, with the corresponding ratios of As (III)/As (V) of 0.45 and 1.02, respectively.

During the cultivation at 20 °C, there was relatively strong mineralization of organic matter, leading to a significant decrease in the Eh of the sediment (Figure 1), thereby increasing the release intensity of As from the sediment. The rise in environmental temperature also promoted the desorption of arsenates on the solid phase of the sediment and increased the diffusion of arsenate ions in the pore water, gradually accumulating and releasing dissolved As concentrations into the pore water and subsequently into the overlying water. After high and low DO cultivation at 20 °C, the mean values of soluble As (III) + As (V) in the pore water were 22.69 μg/L and 55.78 μg/L, respectively, with the corresponding ratios of As (III)/As (V) of 1.20 and 3.84. In the overlying water after high and low DO cultivation at 20 °C, the concentrations of dissolved As(III) + As(V) were 3.83 μg/L and 9.70 μg/L, respectively, with ratios of As (III)/As (V) of 0.46 and 1.67. By comparing the migration and transformation intensities of dissolved As at 5 °C and 20 °C, it can be observed that the conversion and release of sediment As showed a clear temperature-dependent characteristic. Low-temperature conditions had a significant inhibitory effect on the release, migration, and speciation transformation of As in the sediment. Under low-temperature conditions, microbial activity in the sediment was low, and the release rate and biogeochemical transformation rate of As (III) and As (V) were relatively slow [13]. Therefore, regardless of experiencing high or low DO cultivation under low-temperature conditions, both the total As concentrations and As (III)/As (V) ratios were relatively low in the overlying water and pore water.

Under both 5 °C and 20 °C temperature conditions, the concentrations of dissolved As (III) + As (V) under high DO cultivation were lower than those under low DO cultivation, which were consistent with the trends observed for soluble Fe and Mn in the pore water. This indicates that the extent of reduction and dissolution of Fe/Mn-OOH was an important controlling factor for the dissolution of As from sediment, which confirmed the observations of earlier research [47,48,49]. However, during the first two days of cultivation at 5 °C, the mean content of dissolved As (III) + As (V) in the pore water under high DO conditions (3.66–4.68 μg/L) was higher than that under low DO cultivation (2.47–4.13 μg/L), while soluble Fe, Mn, and S showed the opposite trend. This suggests that prior to low-temperature cultivation, the oxidation and decomposition of As sulfide might contribute more to the enhancement of soluble As in sediments [50] compared to the reduction and dissolution of Fe/Mn-OOH. On the 30th day of high DO incubation, the concentrations of soluble As (III) + As (V) decreased synchronously with the contents of dissolved Fe and Mn in the pore water at both 5 °C and 20 °C, indicating that dissolved As might have been adsorbed or co-precipitated by newly formed Fe/Mn-OOH. Conversely, on the 30th day of low DO cultivation, despite a reduction in dissolved Fe and Mn contents at both 5 °C and 20 °C, the average contents of dissolved As (III) and As (V) continued to rise, and the ratios of As (III) to As (V) kept on climbing (Figure 3 and Figure 4). This suggests that the release of As through the reduction and dissolution of Fe/Mn-OOH was not the exclusive source of As in the pore water during low DO cultivation. The reduction of As itself might have become an important source of dissolved As in the later stages of cultivation [18,51]. Given that the reduction product of As, As (III) readily dissociated from the solid phase of sediment under near-neutral conditions and rapidly released into water [52,53], the accumulation of soluble As, particularly As (III), in the pore water gradually occurred under low DO conditions at both 5 °C and 20 °C.

**Figure 6:**
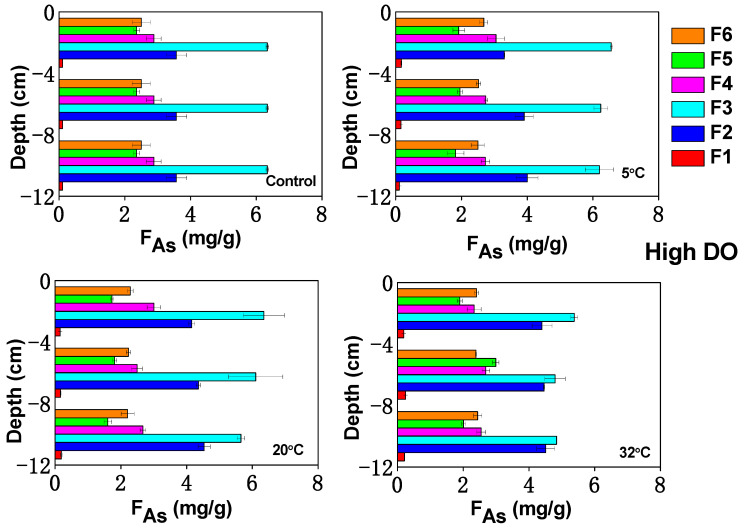

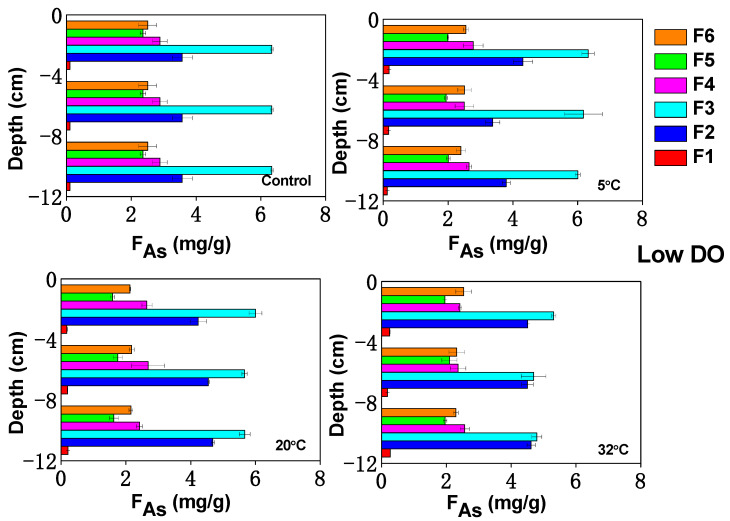
The respective changes of As components (F1: non-specifically adsorbed As, F2: specifically adsorbed As, F3: amorphous Fe/Mn-OOH-As, F4: crystalline Fe/Mn-OOH-As, F5: organically bound As, F6: residual As) in sediments after culture under high and low DO condition.

**Figure 7:**
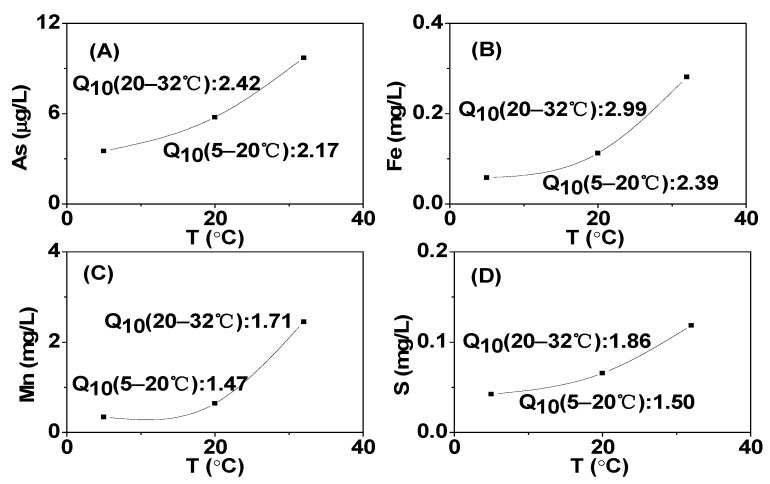
The temperature dependence of sediment As release ((**A**) and related elements (**B**) Fe, (**C**) Mn, and (**D**) S) under low DO culture.

The high-temperature condition of 32 °C had a more significant effect on the release of dissolved As in sediments (Figure 3). With increasing temperature, the rate of organic matter mineralization increased, leading to rapid oxygen consumption in the pore water and more frequent migration and transformation of As at the SWI (Figure 6 and Figure 7). Under both high and low DO conditions, there was an intense release of soluble As in the pore water on the first day of cultivation, with average concentrations of 33.05 μg/L and 44.08 μg/L for dissolved As (III) + As (V), significantly higher than the concentrations during the same period under 5 °C and 20 °C conditions (*p* < 0.01). This indicates that temperature had a more pronounced promoting effect on the desorption of As from the sediment surface compared to DO levels in the initial stage of incubation. The concentrations increased to 3.88 μg/L and 6.26 μg/L in the overlying water, indicating that As in the pore water migrated quickly to the superjacent water under high-temperature levels. From the second to the sixth day of cultivation, under high-temperature conditions, the concentrations of dissolved As (III) + As (V) in the pore water continued to increase under both high and low DO conditions. There was also frequent exchange of As between the pore water and the overlying water (Figure 3 and Figure 5). By the 17th day of incubation, the mean concentrations of soluble As (III) + As (V) in the pore water reached their peak values under both high and low DO conditions (Figure 3). They were 59.98 μg/L and 63.99 μg/L, respectively, which were 4.84 times and 3.03 times higher than the corresponding conditions under 5 °C. The ratios of As (III)/As (V) under high and low DO conditions were 3.79 and 3.83, respectively, which were 3.28 times and 2.22 times higher than the corresponding ratios under 5 °C conditions. The ratio of As (III)/As (V) was positively correlated with Fe and Mn (Table S2).

All of these results indicate that high-temperature conditions were most favorable for the release and speciation transformation of As in sediment, consistent with previous research [11,54]. Previous studies have shown that many microorganisms exhibit maximum activity at 30 °C [55,56], with the strongest reduction capability for As (V) to As (III), leading to an increase in As (III) content and total As concentration [57]. Additionally, temperature might regulate the release of As by modulating the release of organic matter. According to previous research [58,59], it might be attributed to high-temperature conditions accelerated the release of unstable organic matter from sediments, which could be utilized as an energy source by Fe (III) oxide-reducing bacteria and As-reducing bacteria to reduce amorphous Fe (III) oxides and As (V), leading to the release of As from the solid phase to the aqueous phase, thereby causing an increase in As concentration in the pore water. However, on the 30th day of cultivation, there was a significant decrease in the contents of soluble As (III) + As (V) in the pore water and overlying water under conditions of both high and low DO. From the redox potential on the 30th day, the system did not have the conditions to form AsH_3_. The total As content in sediment after high-temperature cultivation (anaerobic: 17.40 μg/g, aerobic: 17.84 μg/g) was significantly lower than the initial total content (18.15 mg/kg) [26], indicating that a large amount of re-adsorption of pore water and overlying water As by the sediment did not occur. At the same time, a marked decline in the concentrations of dissolved Fe, Mn, and S was observed. Therefore, the simultaneous decrease in the total As and dissolved As in the aqueous phase was likely due to the conversion of some dissolved As into colloidal As under high-temperature conditions. Previous studies have shown that a considerable amount of trace metals in water could be strongly adsorbed and complexed by colloids, causing the transformation from free trace metal species to colloidal trace metal species [60]. Under high-temperature conditions, the concentration of colloidal trace metals gradually increased [61]. Colloidal particles are defined by researchers as particles with a size range of 1 kD^−1^ μm [62]. In this study, dissolved As was obtained using a 0.45 μm filter membrane, so it was possible that a considerable amount of As in the aqueous phase existed in the form of colloids under high-temperature conditions, leading to a smaller measured concentration of dissolved As in the high-temperature environment.

Studies have demonstrated a diminished impact of temperature on lower redox conditions and, thus, on the regulation of As migration during shorter time intervals [11,63,64,65]. This finding further supports the notion that the primary determinant governing As migration was its redox potential rather than temperature. Nevertheless, as the duration of observation was prolonged, temperature emerged as an influential factor governing the movement of As within lake systems. This influence was exerted through its effect on the overall redox conditions prevailing in benthic sediments, which was more prominently observed over seasonal scales due to annual temperature variations exceeding 20 °C [66].

The migration of As during incubation was highly influenced by temperature. The reduction of As (V) species and subsequent precipitation of newly formed As (III) species played a crucial role in promoting As migration [67]. This phenomenon can be attributed to the lower adsorption affinity of As (III) species towards sediment particles compared to As (V) species, especially under near-neutral conditions and low to moderate loads. Importantly, there existed a strong correlation between temperature, redox potential, and the production of As (III), suggesting the significant role of microbial metabolism in driving the reduction of As (V) to As (III) [68].

### 3.4. Variation of the Cumulative Release Rate of Each Element in Overlying Water

**Figure 8:**
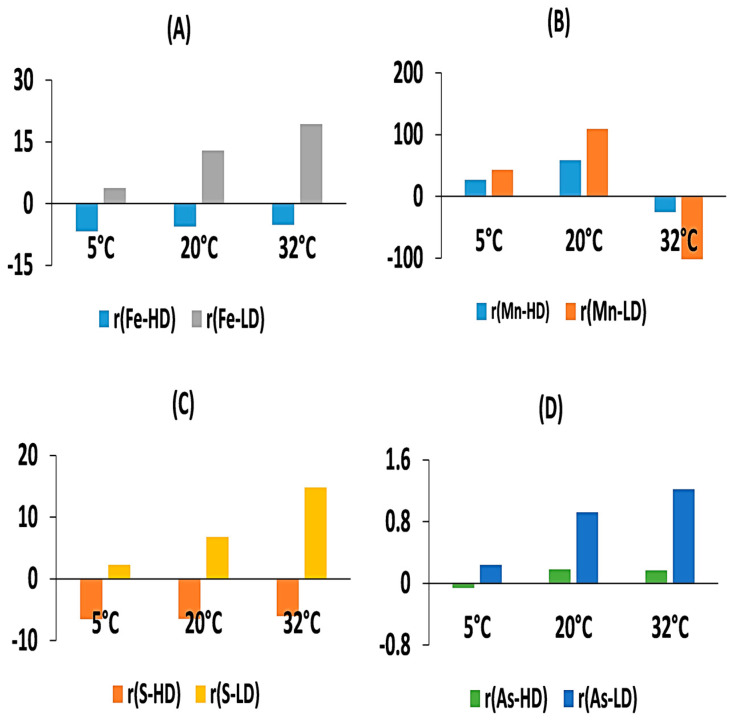
The influence of temperature on the cumulative release rates from overlying water under varying DO levels: (**A**) Fe-high DO(HD), Fe-low DO(LD); (**B**) Mn-HD, Mn-LD; (**C**) S-HD, S-LD; and (**D**) As-HD, As-LD.

According to the data presented in Figure 8, after exposure to high DO levels, both Fe and S in the dissolved state showed signs of being adsorbed, regardless of the temperature condition applied. After low DO cultivation, sediment releases As, Fe, Mn, and S (except for dissolved Mn at 32 °C). Under low DO conditions at 5 °C, 20 °C, and 32 °C, the release rates of As were 0.24 μmol/(m^2^·d), 0.92 μmol/(m^2^·d), and 1.22 μmol/(m^2^·d), respectively. The release rates of Fe were 3.78 μmol/(m^2^·d), 12.90 μmol/(m^2^·d), and 19.31 μmol/(m^2^·d) under the respective temperature conditions. The release rates of Mn were 42.96 μmol/(m^2^·d), 109.45 μmol/(m^2^·d), and −125.55 μmol/(m^2^·d), respectively, except for dissolved Mn at 32 °C. The release rates of S were 2.26 μmol/(m^2^·d), 6.82 μmol/(m^2^·d), and 14.80 μmol/(m^2^·d) respectively. These results indicate that increasing temperature favors the formation of anaerobic conditions, accompanied by strong reduction and release of FeOOH and sulfates, resulting in the migration of a large amount of dissolved As to the water. Under high DO conditions at 32 °C, the migration behavior of Fe, Mn, and S in the overlying water exhibited adsorption behavior, which might also contribute to the absence of a significant increase in the release of As compared to the 20 °C condition after cultivation.

### 3.5. Influence of Temperature on As Fractions in Sediments under Varying DO Conditions

#### 3.5.1. Ion Exchange As

After cultivation under different conditions, the solid-phase As fractions in sediment have changed. The exchangeable As fraction is divided into non-specifically adsorbed As (F1) and specifically adsorbed As (F2), which are the most easily dissociated As fractions in the sediment. The initial contents of F1 and F2 in the sediment were 0.11 μg/g and 3.57 μg/g respectively (Figure 6). After low DO cultivation, the contents of F1 at 5 °C, 20 °C, and 32 °C were 0.16 μg/g, 0.20 μg/g, and 0.24 μg/g, respectively, which were significantly higher than those of the initial sediment. After low DO cultivation, the content of F2 under each temperature condition also showed an increasing trend compared to the initial sediment, and there were significant differences between temperature conditions (*p* < 0.05). Similar to F1, the higher the temperature, the larger the F2 in the sediment. After high DO cultivation, the contents of F1 and F2 also increased, but they were smaller than those of F1 and F2 after low DO cultivation (Figure 6). This was because, after high DO cultivation, the stable form of As (V) existed in the soluble As in the upper layer of the sediment (Figure 3), which was easily adsorbed onto the surfaces of clay minerals, Fe/Mn-OOH and underwent precipitation, leading to the transformation of non-specifically and specifically adsorbed As into more stable As fractions. Therefore, F1 and F2 were relatively small under high DO conditions. A comparison with other temperature conditions showed that F1 and F2 were the largest under high-temperature conditions. This was because high-temperature conditions were more favorable for the activation of sediment, and under high-temperature conditions, the sediment exhibited a decreased Eh (Figure 1), causing the transformation of stable As fractions into unstable fractions. Although increasing the temperature could also accelerate the diffusion of As, leading to the transformation of more F1 and F2 into stable fractions [69], from this study, it is observed that increasing the temperature resulted in a decrease in sediment Eh, which corresponded to a greater tendency for the transformation of As into unstable and easily dissociated fractions.

#### 3.5.2. Iron/Manganese (Hydrogen) Oxide Combined As

Fe/Mn-OOH-bound As can be categorized into amorphous Fe/Mn-OOH-As (F3) and crystalline Fe/Mn-OOH-As (F4). The initial content of F3 and F4 in the sediment were 6.34 μg/g and 2.88 μg/g, accounting for 35.67% and 16.23%, which were the largest As fractions in the sediment and played an important role in the bioavailability of As. After high DO (low DO) cultivation, the mean contents of F3 at 5 °C, 20 °C, and 32 °C were 6.33 (6.17) μg/g, 6.03 (5.77) μg/g, and 5.00 (4.93) μg/g, respectively; while the mean contents of F4 were 2.84 (2.64) μg/g, 2.72 (2.58) μg/g, and 2.52 (2.45) μg/g, respectively. Under both low and high DO conditions, there were significant differences in the content of F3 in the sediment (*p* < 0.05), indicating that temperature had a significant influence on the variation of F3 in the sediment.

From the sediment profile distribution, it can be seen that after high DO cultivation at 5 °C and 20 °C, the contents of F3 and F4 in the upper sediment layer increased. This was likely because high DO conditions favored the transformation of exchangeable As into Fe/Mn-OOH-bound As. In the middle and lower sediment layers, the Eh were relatively low, and F3 and F4 underwent reduction, resulting in varying degrees of decrease. Under low DO cultivation conditions at 5 °C and 20 °C, Fe/Mn-OOH-bound As underwent reduction and dissolution, which resulted in the liberation of As from the sediment.

This decrease in F3 fraction and the increase in As concentration in the pore water were consistent with this inference and were similar to the results reported by Liu et al. [70]. After cultivation at 32 °C, the contents of F3 and F4 in the sediment profile decreased. Under 32 °C conditions, the Eh was the lowest, which was most favorable for the reduction of F3 and F4. Furthermore, under high-temperature conditions, desorption of As from Fe-OOH solid phase was known to be an endothermic reaction [71]. In addition, the biologically mediated reduction and dissolution of Fe-OOH can also be accelerated with increasing temperature [72]. Therefore, compared to other temperature conditions, the decreases in F3 and F4 in the sediment after cultivation at 32 °C were the most significant. Although there was no synchronous increase in dissolved As, Fe, and Mn in the overlying water and pore water, as mentioned in Section 3.3, a considerable amount of As released from F3 and F4 converted to colloidal As in the water.

#### 3.5.3. Organically Bound As

Organically bound As refers to the compounds formed by the binding of As with organic substances. In sediments, organic matter can complex or covalently bind with As, forming organically bound As. In this study, organically bound As is referred to as the F5 fraction.

The presence of DO determined the dominance of aerobic microorganisms over anaerobic microorganisms in the environment, leading to different impacts on the release of As from sediments and influencing the transformation of organically bound As. The F5 fraction mainly consisted of As bound to insoluble substances such as sulfides (FeAsS), clays, and organic matter [73]. Under high DO conditions, the oxygen consumption and degradation of organic matter by microorganisms were more intense [74], thereby causing a rapid decomposition of F5 and a decrease in its content. However, in this experiment, due to the low content of organically bound As in the sediment, re-adsorption of As might occur under microbial activities, resulting in a much lower release of As both in terms of quantity and rate compared to anaerobic conditions (Figure 6).

Under high temperatures and high DO conditions, the solubility of oxygen remained low, so the dominance of aerobic microorganisms was not significant, and their oxidative effect on F5 was minimal. On the other hand, under low DO conditions, anaerobic microorganisms not only consumed sulfate to produce H_2_S but also consumed organic matter [75], converting organically bound As into inorganically bound As and promoting its release. However, high-temperature conditions also accelerated the diffusion of sediment As, leading to fixation with organic matter [76]. Therefore, the average content of F5 decreased the least under 32 °C conditions compared to other temperatures (Figure 6), and F5 decreased to 2.00 μg/g and 2.23 μg/g under low and high DO conditions, respectively.

### 3.6. Controls of Temperature and DO on As Mobility in Sediment–Water Incubations

The desorption of As from the solid-phase Fe/Mn-OOH surfaces in sediment was an important mechanism controlling the migration of As [77]. In the initial stage of low DO cultivation, the sediment rapidly depleted oxygen, leading to the reduction of Mn and Fe, as well as the migration of As. Under high DO conditions, there was sufficient oxygen in the upper sediment layer, providing conditions for the decomposition of sediment organic matter and the oxidation of iron sulfides containing As. This became the primary driving force for the release of As from the upper sediment. Due to the limited penetration depth of DO, the lower sediment layer had a lower redox potential. During cultivation, the reduction of Mn and Fe provided electrons for organic matter decomposition.

The Q_10_ values were employed to further investigate the significant impact of temperature on both microbial respiration and the mobilization rates of redox-sensitive species (Figure 7). From Figure 5, it can be seen that the release rate of As was the fastest during the 2nd to 6th day under low DO conditions, so the Q_10_ analysis was conducted for this period, revealing a strong temperature dependence of As release from the sediment. The Q_10_ values for As, Fe, Mn, and S increased from 5 °C to 20 °C, with values of 2.17, 2.39, 1.47, and 1.50, respectively. As the temperature further increased from 20 °C to 32 °C, the Q_10_ values for As, Fe, Mn, and S increased to 2.42, 2.99, 1.71, and 1.86, respectively. It can be observed that the Q_10_ value of As was synchronized with the Q_10_ values of the other redox-sensitive elements, consistent with previous research findings [72,78]. Fe(III) and Mn oxides were considered important hosts for As in the sediments of Lake Taihu [66], so the reduction dissolution of As-bearing Fe(III) and Mn minerals might also play a role in As migration. The concentration of As was significantly correlated with Fe^2+^ and Mn^2+^ (*p* < 0.01) (Table S2). Therefore, under low DO conditions, with the increase in temperature, the reduction of Fe/Mn-OOH and sulfates was enhanced, leading to a significant promotion of dissolved As. The Q_10_ values of As showed that a small increase in water temperature above about 20 °C might significantly elevate As concentrations in Taihu Lake. The findings suggest that temperature-dependent reduction processes within the hyporheic zone’s benthic sediments played a crucial role in controlling As mobility across various spatial and temporal scales, aligning with our field observations at both local and regional levels [66].

## 4. Conclusions

Temperature and DO had significant effects on the release of sediment-bound As. Through establishing reducing conditions and the close coupling between reduction and the release of As in the solid phase, an increase in temperature enhanced the mobility of As. At a low temperature of 5 °C, there was minimal release of sediment-bound As. However, as the temperature rose, an anaerobic environment was facilitated, promoting the reduction of Fe/Mn-oxides and sulfates, thus facilitating the release and desorption of various fractions and forms of sediment-bound As. Under low DO conditions, the release intensity of sediment-bound As further increased at different temperature conditions. The results from laboratory culturing experiments aligned with observations at the outdoor lake scale, indicating that increased anaerobic microbial metabolism under high-temperature conditions leads to lower oxidation–reduction potential, which became the primary driving factor for As dissolution. Thus, seasonal temperature variations were a key controlling factor in the migration of As in the Lake Taihu sediments. Predictions of climate warming and higher seasonal temperature extremes had significant implications for the migration of sediment-bound As. Water temperature plays a crucial role in seasonal changes of As in aquatic systems due to its regulation of microbial activities.

## Data Availability

The data presented in this study are available on request from the corresponding author. The data are not publicly available due to the requirements of the research institution.

## Supplementary Materials

The following supporting information can be downloaded at: https://www.mdpi.com/article/10.3390/toxics12070471/s1, Figure S1: The effect of temperature on dissolved Fe in pore water under different DO conditions: high DO: (A) 5 °C, (B) 20 °C and (C) 32 °C; low DO: (D) 5 °C, (E) 20 °C and (F) 32 °C; Figure S2: The effect of temperature on dissolved Mn in pore water under different DO conditions: high DO: (A) 5 °C, (B) 20 °C and (C) 32 °C; low DO: (D) 5 °C, (E) 20 °C and (F) 32 °C; Figure S3: The effect of temperature on dissolved S of pore water under different DO conditions: high DO: (A) 5 °C, (B) 20 °C and (C) 32 °C; low DO: (D) 5 °C, (E) 20 °C and (F) 32 °C; Table S1: The linear equation of the standard regression curve, the correlation coefficient and LODs of each element. Table S2: The correlation analysis between the mean values of dissolved As and dissolved Fe, Mn and S in sediment.
