# Supplementary material for: Temperature and Dissolved Oxygen Drive Arsenic Mobility at the Sediment—Water Interface in the Lake Taihu
Source: Toxics. 2024 Jun 29;12(7):471. doi: 10.3390/toxics12070471 (PMC11281122; doi:10.3390/toxics12070471)
Supplement: Supplementary file 1 [file toxics-12-00471-s001.zip › toxics-3032411-supplementary.pdf]

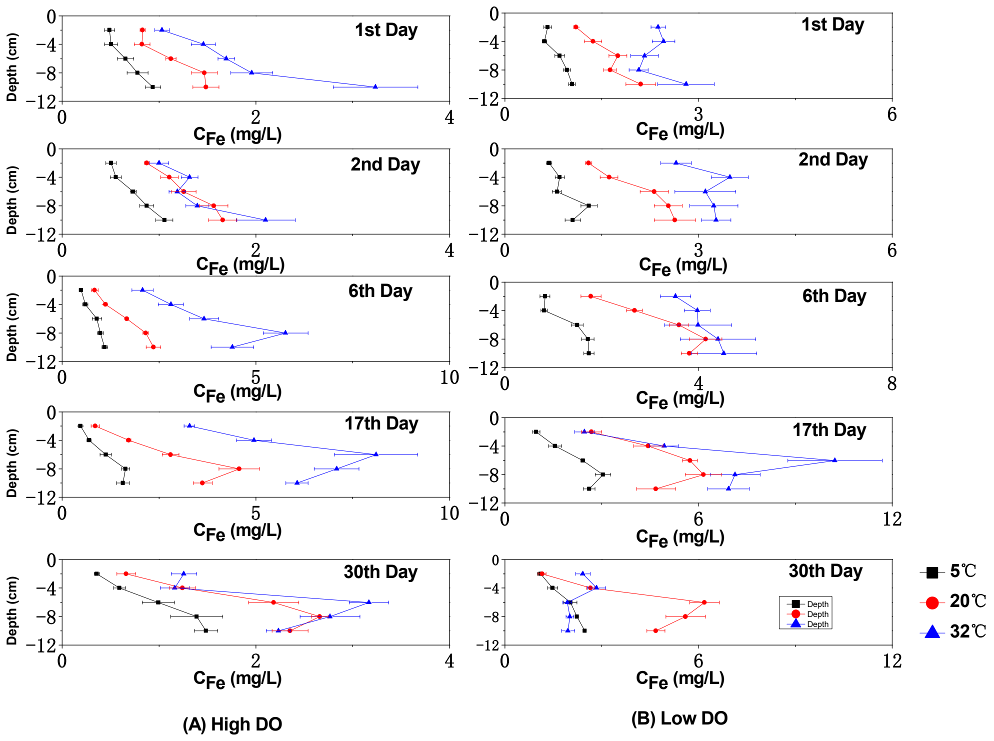

Figure S1 The effect of temperature on dissolved Fe in pore water under different DO conditions: (**A**) high DO; (**B**) low DO.

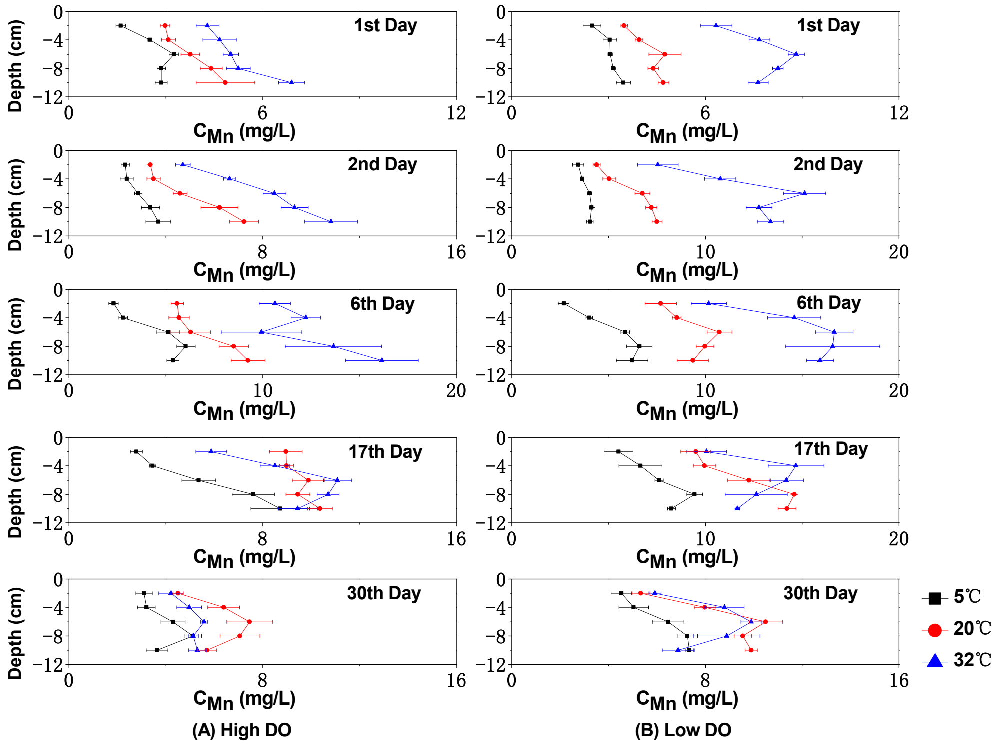

Figure S2 The effect of temperature on dissolved Mn in pore water under different DO conditions: (**A**) high DO; (**B**) low DO.

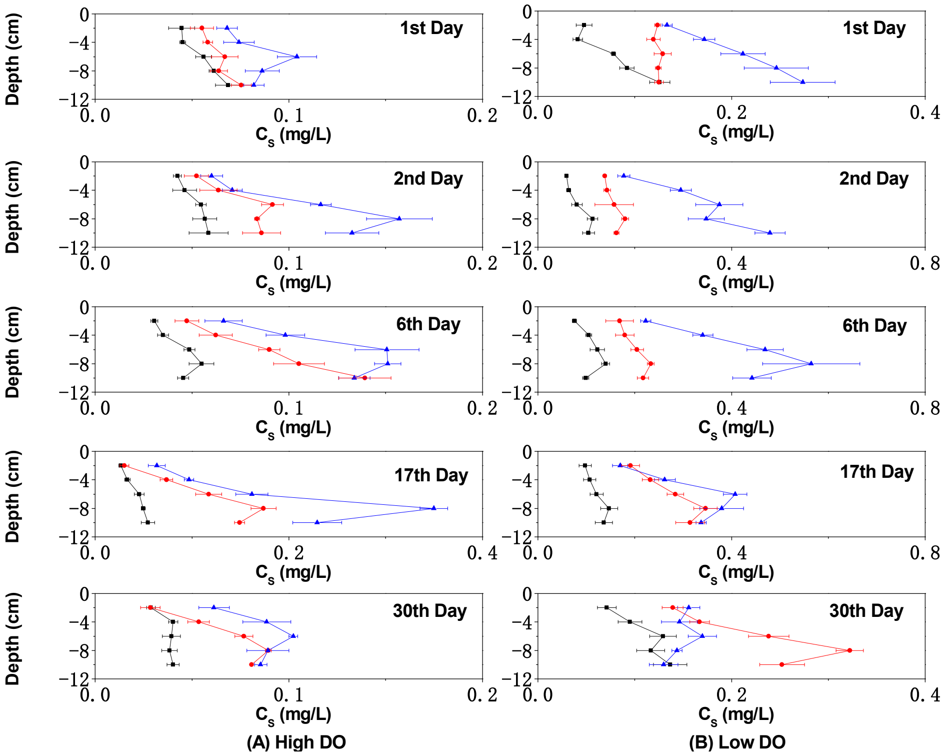

Figure S3 The effect of temperature on dissolved S of pore water under different DO conditions: (**A**) high DO; (**B**) low DO.

Table S1 The linear equation of the standard regression curve, the correlation coefficient and LODs of each element

**Detection object**	**Linear equation**	**r**	**LOD**	**Measurement method**
As	y = 0.0302x + 0.0014	0.9997	0.02 μg/L	ICP-MS
As (V)	y = 0.7359x − 0.000055	0.9998	0.06 μg/L	HPLC-ICP-MS
As (III)	y = 0.6908x − 0.000069	0.9999	0.05 μg/L	HPLC-ICP-MS
Fe	y = 3.624x + 0.1607	0.9998	0.8 μg/L	ICP-MS
Mn	y = 0.1765x + 0.1763	0.9997	0.36 μg/L	ICP-MS
Fe (II)	y = 0.2028x + 0.0092	0.9997	0.04 mg/L	Phenanthroline colorimetry
S (II)	y = 0.1538x + 0.0014	0.9996	0.006 mg/L	Methylene blue method

Table S2 The correlation analysis between the mean values of dissolved As and dissolved Fe, Mn and S in sediment

	High DO	Low DO
5 °C	$C_{Fe} = 0.0591\ C_{As} + 0.406$	$C_{Fe} = 0.0658\ C_{As} + 0.7148$
	$R^2 = 0.669$ ($P < 0.01$)	$R^2 = 0.7845$ ($P < 0.01$)
	$C_{Mn} = 0.2408\ C_{As} + 2.0158$	$C_{Mn} = 0.1737\ C_{As} + 3.271$
	$R^2 = 0.5044$ ($P < 0.01$)	$R^2 = 0.7104$ ($P < 0.01$)
	$C_S = -9E^{-05}\ C_{As} + 0.0475$	$C_S = 0.0022\ C_{As} + 0.0778$
	$R^2 = 0.0016$ ($P < 0.01$)	$R^2 = 0.4541$ ($P < 0.01$)
20 °C	$C_{Fe} = 0.0724\ C_{As} + 0.4089$	$C_{Fe} = 0.0684\ C_{As} + 0.9141$
	$R^2 = 0.7049$ ($P < 0.01$)	$R^2 = 0.7966$ ($P < 0.05$)
	$C_{Mn} = 0.1683\ C_{As} + 3.2731$	$C_{Mn} = 0.1103\ C_{As} + 4.5119$
	$R^2 = 0.649$ ($P < 0.05$)	$R^2 = 0.577$ ($P < 0.01$)
	$C_S = 0.002\ C_{As} + 0.0442$	$C_S = 0.0025\ C_{As} + 0.1133$
	$R^2 = 0.4053$ ($P < 0.01$)	$R^2 = 0.6692$ ($P < 0.01$)
32 °C	$C_{Fe} = 0.06\ C_{As} + 0.5291$	$C_{Fe} = 0.0561\ C_{As} + 0.856$
	$R^2 = 0.3329$ ($P < 0.01$)	$R^2 = 0.3578$ ($P < 0.01$)
	$C_{Mn} = 0.1072\ C_{As} + 3.5542$	$C_{Mn} = 0.0924\ C_{As} + 6.4489$
	$R^2 = 0.3932$ ($P < 0.01$)	$R^2 = 0.3219$ ($P < 0.01$)
	$C_S = 0.0022\ C_{As} + 0.0262$	$C_S = 0.004\ C_{As} + 0.0829$
	$R^2 = 0.4267$ ($P < 0.01$)	$R^2 = 0.4421$ ($P < 0.01$)
